# Determinants of Uterine Rupture and Its Management Outcomes among Mothers Who Gave Birth at Public Hospitals of Tigrai, North Ethiopia: An Unmatched Case Control Study

**DOI:** 10.1155/2020/8878037

**Published:** 2020-10-28

**Authors:** Meresa Berwo Mengesha, Desta Abraha Weldegeorges, Yared Hailesilassie, Weldu Mammo Werid, Mulu Gebretsadik Weldemariam, Fissaha Tekulu Welay, Senait Gebreslasie Gebremeskel, Berhanu Gebresilassie Gebrehiwot, Hagos Degefa Hidru, Hirut Teame, Haftay Gebremedhin, Natnael Etsay Assefa

**Affiliations:** ^1^Department of Midwifery, Adigrat University, College of Medicine and Health Sciences, Adigrat, Tigrai, Ethiopia P.O. Box No. 50; ^2^Department of Nursing, Adigrat University College of Medicine and Health Sciences, Adigrat, Tigrai, Ethiopia; ^3^Department of Public Health, Adigrat University College of Medicine and Health Sciences, Adigrat, Tigrai, Ethiopia

## Abstract

**Introduction:**

Uterine rupture is a leading cause of maternal death in Ethiopia. Despite strengthening the health care system and providing basic and comprehensive emergency obstetric care closer to the communities, uterine rupture continues to produce devastating maternal and fetal outcomes. Although risk factors of uterine rupture are context specific, there is lack of clarity in our context towards the contributing factors and untoward outcomes of uterine rupture. This study was conducted to identify the risk factors of uterine rupture and its impacts in public hospitals of Tigrai.

**Objective:**

This study would identify determinant factors of uterine rupture and its management outcomes among mothers who gave birth in public hospitals in Tigrai region, North Ethiopia.

**Method:**

A retrospective hospital-based unmatched case control study design was implemented with 135 cases of women with uterine rupture and 270 controls of women without uterine rupture. Cases were enrolled consecutively from case notes of women who gave birth from 1/9/2015 to 30/6/2019, while charts (case note) of women without uterine rupture found following the cases were selected randomly and enrolled. Bivariate and multivariate logistic regression with 95% confidence interval was used to identify the determinants of uterine rupture.

**Result:**

Mothers referred from remote health institutions (AOR 7.29 (95% CI: 2.7, 19.68)), mothers who visited once for antenatal care (AOR 2.85 (95% CI: 1.02, 7.94)), those experiencing obstructed labor (AOR 13.33 (95% CI: 4.23, 42.05)), and birth weight of a newborn greater than four kilograms (AOR 5.68 (95% CI: 1.39, 23.2)) were significantly associated with uterine rupture. From 135 mothers who develop uterine rupture, 13 (9.6%) mothers died and 101 (74.8%) fetuses were stillborn. Obstetrical complications like abdominal hysterectomy in 75 (55.6%) of mothers and excessive blood loss in 84 (57.8%) were additional untoward outcomes of uterine rupture.

**Conclusion:**

Referrals from remote health institutions, once-visited antenatal care, obstructed labor, and birth weight of newborns greater than four kilograms were significant determinants of uterine rupture. Maternal death, stillbirth, hysterectomy, and hemorrhage were adverse outcomes. The findings of this study suggest early identification of factors that expose to uterine rupture during antenatal care, labor, and delivery must be attended to and further prospective studies are needed to explore predictors of untoward outcomes. Knowing the determinants of uterine rupture helps prevent the occurrence of a problem in pregnant women, which reduces maternal morbidity and mortality, and would have a tremendous help in identifying the best optional strategies in our current practices. This assertion was added to the abstract concluding session.

## 1. Introduction

Uterine rupture is a disruption of the uterine wall during pregnancy or childbirth. Usually, destruction to the uterus is not correctable and the outcome is often a hysterectomy [1]. Uterine rupture is a devastating obstetric condition that put the life of the mother and the baby at risk [2]. Its magnitude is greater in Asia and Africa than in high-income countries [3]. The incidence of uterine rupture in Africa ranges from 0.5% to 9.5% of births [4–7].

In Ethiopia, the prevalence of uterine rupture ranges from 1.244% to 9.5% [4, 7, 8]. Although the magnitude is relatively low, it accounts for 18.8% to 36% of maternal mortality [9] and more than 35% of registered maternal deaths were due to uterine rupture [4].

Mothers experiencing uterine rupture outcomes range from 3% to 12.3% vesicovaginal fistulas, 6.1% rectovaginal fistulas, and 16% bladder ruptures; of them were complication of management of ruptured uterus. Direct complication of ruptured uterus includes 59.8% to 88.8% which incur severe blood loss; and 14% to 51.8% undergo total abdominal hysterectomy. The magnitude of fetal mortality is very high with 1.7% to 7% of babies surviving after uterine rupture; 93% to 98.3% of them were stillbirths [4, 5, 7]. Though determinant factors for uterine rupture differ across localities due to differences in sociodemographic status, readiness and ease of access to skilled birth attendants, and health system efficacy, previous studies have found that labor induction, grand multiparity, lack of ANC follow-up, history of previous caesarian section (C/S), prolonged labor, obstructed labor, lack of partograph utilization, and instrumental delivery were significantly associated with uterine rupture [2, 5, 8, 10–13].

Despite strengthening the health care system and provision of basic and comprehensive emergency obstetric care, Ethiopian women continue to face devastating maternal and fetal outcomes, particularly in the study area [14]. This study is aimed at addressing determinant factors of uterine rupture and its adverse maternal and fetal management outcomes in public hospitals of Tigrai. Knowing the risk factors of uterine rupture will potentially assist women, providers, and health systems to take actions on each factor to decrease maternal as well as perinatal morbidity and mortality related to uterine rupture.

## 2. Methods and Materials

### 2.1. Study Design and Setting

A retrospective hospital-based unmatched case control study design was implemented at public hospitals in Tigrai region, from cards (case notes) of mothers who gave birth from 1/9/2015 to 30/6/2019. Cards of mothers, who gave birth from 1/9/2015 to 30/6/2019 in selected public hospitals of Tigrai, were retrieved. We have used an unmatched case control study for frequency and ensured that cases and controls are not identical; however, they are comparable and share the same geographical and social backgrounds. In addition, we have tried to avoid seasonal impact. For example, during rainy seasons, women in rural areas do not come to seek obstetric care at a higher facility due to the unavailability of transportation.

### 2.2. Selection of Cases

Uterine rupture was defined as tearing of the uterine wall either partially or completely during pregnancy and labor, diagnosed clinically and later confirmed at laparotomy by the attending physician. The cases were obtained from the labor and delivery ward, operating theatre registers, and from the patients' case files retrospectively. Chart number of women diagnosed with uterine rupture who met the criteria was enrolled consecutively.

### 2.3. Selection of Controls

Controls were women who had spontaneous vaginal delivery or who delivered by caesarean section without uterine rupture as a complication. Charts (case notes) of women without uterine rupture (control) found after the cases (since cases and controls should be comparable regardless of the presence of the disease of interest, we enrolled controls who were admitted following the cases to avoid seasonal impact on transportation from rural areas and other parameters) were selected randomly and enrolled.

### 2.4. Inclusion Criteria

Cases are all mothers diagnosed with uterine rupture during pregnancy and labor and delivery in selected public hospitals of Tigrai.

Controls are all mothers who gave birth without experiencing uterine rupture in selected public hospitals of Tigrai.

### 2.5. Exclusion Criteria

If the mother's card (case note) missed dependent and other significant variables under study, then it will be excluded from the study; missed and tear cards were excluded.

### 2.6. Sample Size Determination

Sample size was calculated using Epi-info Version 7 based on the following assumptions: 95% level of confidence, 80% power, taking two to one ratio of controls to case (2 : 1). The proportion of control with educational level of primary school is 67% and the proportion of case with educational level of primary school is 80.63%, with the odds ratio of primary school educated women as 2.05 times more likely to develop uterine rupture [13]. The final sample size was 135 cases and 270 controls. There were 14 incomplete cards (missing essential variables and discarded (tear cards)), and 6 case notes (patient cards) were lost. We have used 5% contingency for the incomplete and missed patient's cards, while our final complete records for both cases and controls were 405.

### 2.7. Sampling Procedure

**Figure 1:**
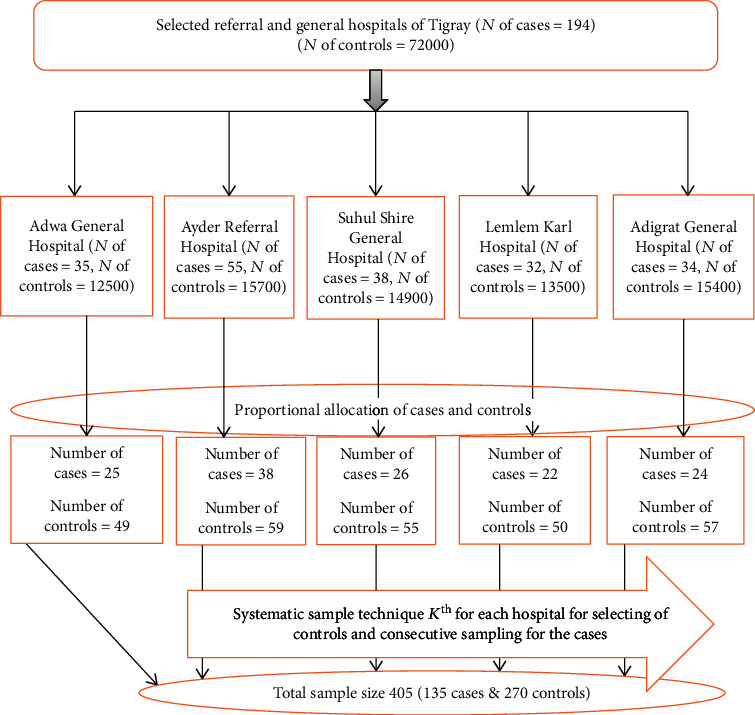
Schematic presentation of sampling technique to identify determinants of uterine rupture and management outcomes among mothers who give birth in public hospitals of Tigray, 2018/2019.

This study was conducted in selected public hospitals in Tigrai. There are 14 general hospitals and two referral hospitals in Tigrai. One referral and four general hospitals were selected randomly from all general and referral hospitals found in Tigrai region. Ayder Referral Hospital, Lemlem Karl General Hospital, Adigrat General Hospital, Adwa General Hospital, and Suhul General Hospital were selected. After calculating the previous five years' admission of mothers in obstetric ward and knowing the total case load in each selected hospital, the sample size was allocated to the hospitals proportionally. In the five years' survey, there were 72000 deliveries without uterine rupture (control) and 194 cases in Adwa General Hospital (*N* of cases = 35, *N* of controls = 12500), Ayder Referral Hospital (*N* of cases = 55, *N* of controls = 15700), Suhul Shire General Hospital (*N* of cases = 38, *N* of controls = 14900), Lemlem Karl Hospital (*N* of cases = 32, *N* of controls = 13500), and Adigrat General Hospital (*N* of cases = 34, *N* of controls = 15400). Charts (case note) of women diagnosed with uterine rupture who met the criteria were enrolled consecutively, while charts (case note) of women without uterine rupture (control) found following the cases were selected randomly and enrolled. Given that uterine rupture is rare, we have enrolled charts of women who have met the inclusion criteria until the total sample size was attained (Figure 1).

### 2.8. Study Variables

The uterine rupture was a dependent variable. Sociodemographic factors claimed in the literatures to determine uterine rupture were maternal age, occupation, referral status, residence, and pregnancy and labor and delivery-related factors including labor induction, grand multiparity, lack of ANC follow-up, history of previous caesarian section (C/S), prolonged labor, obstructed labor, lack of partograph utilization, and instrumental delivery.

### 2.9. Data Collection Procedure

Data were collected using a structured checklist adapted from the literature, selecting data from delivery registers, operating theatre registers, and patients' case files, which include sociodemographic variables, pregnancy condition variables, labor and delivery variables, and maternal and fetal management outcomes [2, 4, 5, 13, 15]. Five data collectors with a Bachelor of Science in Midwifery degree were recruited. Wording and consistency of the checklist were corrected after a pretest was done.

### 2.10. Data Analysis

The data were entered into Epi data Version 3.5.1 and exported to the Statistical Package for the Social Sciences (SPSS) Version 20 software for further analysis. Descriptive statistics were presented. The odds ratio was with their 95% confidence interval; two-tailed *P* value was computed to declare the level of significance. Bivariate and multivariate logistic regressions with 95% confidence interval were used to identify determinant factors of uterine rupture. Variables with a *P* value < 0.2 at the bivariate logistic regression were entered to multivariable logistic regression to identify the independent predictors of uterine rupture, to control the confounding variables, and to produce adjusted odds ratio with their corresponding confidence limits. Before performing the multivariable logistic regression, we tested the presence of multicollinearity between independent variables and no multicollinearity was detected. Then, finally, statistical significance was declared if *P* value < 0.05. The goodness of fit of the model was checked by the Hosmer-Lemeshow test. Finally, health facility, number of antenatal visits, experience of obstructed labor, and birth weight of newborn were found to be statistically associated with uterine rupture.

## 3. Results

There were 72000 live births in the study area with 194 cases of uterine rupture in five years' data extraction from case notes of mothers. Given that, the incidence of uterine rupture was 194 in 72000 live births (26.9 in 10000 live births) in the study area.

### 3.1. Sociodemographic-Related Factors

Four hundred and five mother's cards (case notes) were reviewed based on the sampling of 135 cases and 270 controls. The median age of the women in cases and controls was 30 (IQR = 9) and 26 (IQR = 8), respectively. One hundred and thirty (96.3%) of the cases and 267 (98.9%) of the controls have Tigrai ethnicity, eighty (59.3%) of the cases and 138 (51.1%) of the controls were housewives, and 109 (80.7%) of the cases and 249 (92.2%) of the controls were Orthodox Tewahedo believers. One hundred and eight (80%) and 67 (24.8%) of the mothers were referred from remote health facilities aligned with cases and controls, respectively (Table 1).

### 3.2. Obstetric-Related Factors

Forty (29.6%) of the cases and 35 (13%) of the controls were grand multiparous (≥5 births). The proportion of mothers who did not engage in antenatal care in the cases and controls was 22 (16.3%) and 13 (4.8%), respectively. For those who visited antenatal care, 49 (43.8%) of the cases and 170 (64.45%) of the controls had four or more visits. The proportion of women who had previous caesarean delivery in the cases and controls was 26 (19.3%) and 7 (2.6%), respectively, while 16 (11.9%) of the cases and 2 (0.7%) of the controls, who had previous caesarean delivery, had interpregnancy intervals of less than twelve months. Among cases, it was found that 15 (11.1%) of prehemoglobin maternal case group levels were below 7 g per dl while 19 (7%) in the control group were anemic (Table 2).

### 3.3. Labor- and Delivery-Related Factors

For 127 (94.1%) of the cases and 269 (99.6%) of the controls, delivery was at one of the hospitals. Twenty-one (15.6%) of the cases and 14 (5.2%) of the controls began their labor spontaneously. In 118 (87.4%) of the cases and 61 (22.6%) of the controls, labor was not followed by partograph. Sixteen (11.9%) of the cases and 1 (0.4%) of the controls had more than eighteen hours of labor. The proportion of mothers who experience obstructed labor among the case group was 80 (59.3%) and 28 (10.4%) in the control group. Five (3.7%) of the cases and 12 (4.4%) of the controls were instrument deliveries. Among cases, it was found that 29 (21.5%) of newborn birth weights were four kilograms as opposed to 18 (6.7%) in the control group (Table 3).

### 3.4. Factors Associated with Uterine Rupture (Bivariate and Multivariate Analyses)

In bivariate logistic regression, 20 variables showed association with uterine rupture at *P* value of <0.2. In multivariate logistic regression, four variables were significantly associated with uterine rupture at *P* value < 0.05.

The mothers referred from remote health institutions were 7.29 times more likely to develop uterine rupture compared to those who did not have referrals (AOR 7.29; 95% CI: 2.7, 19.68). Mothers who had only one prenatal care visit were 2.85 times more likely to develop uterine rupture compared to those who had four visits or more antenatal care visits with AOR 2.85 (95% CI: 1.02, 7.94). The odds of developing uterine rupture for women experiencing obstructed labor were 13.33 times higher compared to those who had no experience with obstructed labor (AOR 13.33; 95% CI: 4.23, 42.05). Those whose birth weight of newborns was four and above kilograms were 5.68 times more likely to have uterine rupture than those who had newborns less than four kilograms (AOR 5.68; 95% CI: 1.39, 23.2) (Table 4).

### 3.5. Maternal and Fetal Outcomes of Uterine Rupture

Out of 135 mothers who develop uterine rupture, intraoperative findings found that 75 (55.5%) had a complete uterine rupture. With respect to the rupture location, 47 (34.8%) were anterior, 53 (39.25%) posterior, and 35 (25.92%) lateral. Total abdominal hysterectomy was done in 47 (34.8%) of the women, subtotal hysterectomy in 28 (20.74%), uterine repair with bilateral tubal ligation (BTL) in 26 (19.25%), and uterine repair without BTL in 34 (25.2%). Forty-eight (11.9%) of the cases had postoperative hemoglobin value (HGB) of <7 g per dl; 34 (8.4%) cases have HGB value of 7-11 g/dl, and 53 (13.1%) cases have postoperative HGB value of >11 g/dl. Among those who had uterine rupture, 48 (11.9%) of the mothers received blood transfusions.

Thirteen (9.6%) mothers with uterine rupture died secondary to different immediate causes. The documented immediate causes of maternal deaths were hypovolemic shock [8], septic shock [2], and other causes like pulmonary edema [1] and acute renal failure [1]. Among those who had uterine rupture, 101 (74.8%) of their newborns were stillborn (Table 5).

## 4. Discussion

This unmatched case control study is aimed at identifying the risk factors of uterine rupture and describing maternal and fetal outcomes of uterine rupture. The study identified referral from health facility, number of antenatal care visits, experienced obstructed labor, and birth weight of newborn to be significantly associated with uterine rupture. This study also found maternal death, excessive blood loss, abdominal hysterectomy, and a significant number of stillbirths as untoward outcomes of uterine rupture.

This study identified an association between referral status and uterine rupture. Similar to this study, referred mothers from remote health institutions were associated with uterine rupture in Arbaminch (Southern Ethiopia), Mbarara, Uganda, and Debre Markos, Ethiopia [7, 13, 16]. 80% of the cases were referred. The reasons for this may be lack of capacity to recognize and manage abnormal pattern of labor at district, primary hospitals and health centers; despite the government's health policy that envisioned decentralizing emergency and comprehensive obstetric services to the community, still many women referred to referral and tertiary hospitals. This may be due to delays in reaching health facilities due to long distances and poor road networks; many mothers end up with uterine rupture.

Studies from Sihul Shire, Ethiopia, Mizan Tepi, Ethiopia, and Mbarara, Uganda [2, 13, 17] have shown that uterine rupture is highly related with antenatal care attendance, consistent with the findings of this study. The benefit of multiple antenatal visits (recommended four visits) may be contributed through identifying, in advance, maternal risks to rupture, screening for congenital anomalies of fetus, fetal weight, uterine congenital anomalies, malpresentation, and malposition. Therefore, those who have once visited antenatal care may be overlooked in the identification of risk factors contributing to obstructed labor or delaying access to care alternatives (such as caesarian section).

Studies from Sihul Shire (Ethiopia), Mizan Tepi (Ethiopia), and rural Uganda revealed that obstructed labor was a significant risk factor for uterine rupture [2, 5, 17]. This study also showed obstructed labor to be the strongest significant risk factor for uterine rupture. More than half (59.3%) of the cases had obstructed labor. This is often the case in the sense of unsupervised or undersupervised labor in poorly equipped settings, failing detection of prolonged labor by partograph, inadequacy of skilled care providers to detect and give timely, vigilant management for fetal-pelvic disproportion, and overlooked obstructed labor which may lead to rupture due to exhaustion of uterine layers' integrity.

In line with a study conducted in Debre Markos (Ethiopia), France, and Denmark [4, 18, 19], this finding found that those who had a birth weight of four and above kilograms had high odds of developing uterine rupture. The reason might be failing of detecting fetal macrosomia during antenatal care which contributes to fetal-pelvic disproportion and may lead to prolonged and neglected obstructed labor. Though fetal macrosomia is diagnosed retrospectively after birth, antenatal surveillance is mandatory.

This study revealed that hysterectomy had been performed in more than half of the women who develop uterine rupture. This is consistent with the studies from Debre Markos and Nigeria [4, 15] but in discordant with a study from Turkey [20]. The possible explanation could be the differences in health care provider's skills, severity of cases, time for securing hemostasis, the need for fertility and individualized decision-making, and protocols. Among those who had uterine rupture, 48 (11.9%) of the mothers had received blood transfusion. However, blood transfusions were very common in studies done in Debre Markos (78%) and Pakistan (83%) [4, 21]. Such variance may relate to the differences in the hemodynamic state of patients and the availability of blood for transfusion. In the current study, 13 (9.6%) of the mothers with uterine rupture died secondary to different immediate causes, and among those who had uterine rupture, 101 (74.8%) of the newborns were stillbirths. This finding is consistent with studies done in Yirgalem (Southern Ethiopia), Debre Markos (Ethiopia), Mizan Tepi, Uganda, Nigeria, and Yemen [4, 5, 15, 17, 22, 23]. The possible explanations could be due to the absence of antenatal care follow-up, distances hindering referral and increasing time to care, contribution of delays from family, and delays in health institutions.

## 5. Conclusions

In conclusion, this study found that referrals from remote health institutions, antenatal care visit once, obstructed labor, and birth weight of newborns were significant determinants of uterine rupture. Maternal death, stillbirth, hysterectomy, and excessive blood loss were adverse outcomes of uterine rupture.

## 6. Recommendations

The early and timely referral of cases should be promoted for rural and remote health institutions. Health care providers should encourage mothers to complete the recommended four visits as these contribute to full risk assessment and screening opportunities for the mom and the fetus. Labor and delivery should be supervised by trained health care provider, enabling timely and early detection of prolonged labor by partograph; early identification of fetal macrosomia during antenatal or early labor by ultrasound or other clinical methods of predicting fetal weight should be recommended. To this end, preventive strategies for obstructed labor require a multidisciplinary approach, as the factors are multifactorial. In the short-term plan, assessing and identifying high-risk mothers are mandatory. In the long term, promoting adequate dietary diversity and improving nutritional status at household level, empowering and educating women to access a good health care, avoiding harmful traditional practices, access to skilled care during pregnancy and childbirth, i.e., risk assessment during antenatal care, and close monitoring and surveillance of fetomaternal conditions during intrapartum care by utilizing partograph appropriately will benefit to reduce obstructed labor and to prevent maternal death secondary to uterine rupture.

## 7. Limitations

The retrospective nature of the study might miss some sociodemographic and socioeconomic variables despite vigorous tracing in the case file, operation room theatre registration, delivery registration books and neonatal cards, and case file. Further prospective studies are needed to identify predictors of uterine rupture and predictors with untoward management outcomes.

## Figures and Tables

**Table 1:** Sociodemographic characteristics of cases and controls who gave birth at public hospitals of Tigrai, North Ethiopia.

Variables	Category	Cases	Controls	Total
Age	≤19	6 (4.4)	17 (6.3)	23 (5.7)
	20-34	95 (70.4)	211 (78.1)	306 (75.5)
	≥35	34 (25.2)	42 (15.6)	76 (18.8)
Ethnicity	Tigray	130 (96.3)	267 (98.9)	397 (98)
	Afar	5 (3.7)	3 (1.1)	8 (2)
Occupation	Government employee	9 (6.7)	22 (8.1)	31 (7.7)
	Housewife	80 (59.3)	138 (51.1)	218 (53.8)
	Merchant	18 (13.3)	67 (24.8)	85 (21)
	Private employee	16 (11.9)	23 (8.5)	39 (9.6)
	Student	12 (8.9)	20 (7.4)	32 (7.9)
Religion	Orthodox Tewahedo	109 (80.7)	249 (92.2)	358 (88.4)
	Muslim	15 (11.1)	10 (3.7)	25 (6.2)
	Others^∗^	11 (8.1)	11 (4.1)	22 (5.4)
Referral status	Yes	108 (80)	67 (24.8)	175 (43.2)

^∗^Catholic and Protestant. Housewife: a married woman, whose main occupation is caring for her family, managing household affairs, and doing housework.

**Table 2:** Obstetric conditions of cases and controls who gave birth at public hospitals of Tigray, North Ethiopia.

Variables	Category	Cases *N* = 135 (%)	Controls *N* = 270 (%)	Total *N* = 405 (%)	COR (95% CI)
Parity	1-4	95 (70.4)	235 (87)	330 (81.5)	1
	≥5	40 (29.6)	35 (13)	75 (18.5)	2.83 (1.69, 4.72)
Antenatal follow-up	No	22 (16.3)	13 (4.8)	35 (8.6)	3.85 (1.87, 7.91)
	Yes	113 (83.7)	257 (95.2)	370 (91.4)	1
Number of antenatal visits	1	12 (10.7)	22 (8.3)	34 (9)	1.89 (0.88, 4.09)
	2-3	51 (45.5)	72 (27.3)	123 (32.7)	2.46 (1.52, 3.97)
	≥4	49 (43.8)	170 (64.4)	219 (58.2)	1
Gestational age in weeks	28-37	44 (32.6)	60 (22.2)	104 (25.7)	1
	37-42	82 (60.7)	198 (73.3)	280 (69.1)	0.56 (0.35, 0.9)
	≥42	9 (6.7)	12 (4.4)	21 (5.2)	1.02 (0.39, 2.64)
Number of pregnancy when ruptured	Singleton	135 (100)	263 (97.4)	398 (98.3)	1
	Multiple	0	7 (2.6)	7 (1.7)	NA
Previous caesarean delivery	Yes	26 (19.3)	7 (2.6)	33 (8.1)	8.96 (3.78, 21.26)
Interpregnancy interval after CS	Less than 12 months	16 (11.9)	2 (0.7)	18 (4.4)	4.83 (1.61, 14.45)
	Above 12 months	10 (7.4)	5 (1.9)	15 (3.7)	1
Chorioamnionitis	Yes	4 (3)	1 (0.4)	5 (1.2)	8.21 (0.91, 74.22)
Prehemoglobin level	≤7	15 (11.1)	19 (7)	34 (8.4)	2.68 (1.29, 5.56)
	7-11	54 (40)	27 (10)	81 (20)	6.79 (3.96, 11.62)
	≥12	66 (48.9)	224 (83)	290 (71.6)	1

NA = not applicable because of few in numbers. Prehemoglobin level: the level of hemoglobin before uterine rupture.

**Table 3:** Labor and delivery distributions of cases and controls who gave birth at public hospitals of Tigrai, North Ethiopia.

Variable	Category	Cases *N* = 135	Controls *N* = 270	Total *N* = 405	COR (95% CI)
Place of delivery	Health center	8 (5.9)	1 (0.4)	9 (2.2)	16.94 (2.09, 130.09)
	Hospital	127 (94.1)	269 (99.6)	396 (97.8)	1
Partograph use	No	118 (87.4)	61 (22.6)	179 (44.2)	23.78 (13.23, 42.60)
	Yes	17 (12.6)	209 (77.4)	226 (55.6)	1
Duration of labor	More than 18 h	16 (11.9)	1 (0.4)	17 (4.2)	10.19 (1.32, 78.54)
Obstructed labor	Yes	80 (59.3)	28 (10.4)	108 (26.7)	12.57 (7.47, 21.15)
Malpresentations	Yes	18 (13.3)	17 (6.3)	35 (8.6)	0.44 (0.21, 0.88)
Instrumental delivery	No	130 (96.3)	258 (95.6)	388 (95.8)	1
	Yes	5 (3.7)	12 (4.4)	17 (4.2)	1.51 (0.48, 4.77)
Labor started spontaneously	No	21 (15.6)	14 (5.2)	35 (8.6)	3.37 (1.65, 6.86)
	Yes	114 (84.4)	256 (94.8)	370 (91.4)	1
Trial of labor after CS	No	122 (90.4)	267 (98.9)	389 (96)	1
	Yes	13 (9.6)	3 (1.1)	13 (9.6)	9.48 (2.65, 33.89)
Congenital anomaly of baby	No	126 (93.3)	267 (98.9)	393 (97)	1
	Yes	9 (6.7)	3 (1.1)	12 (3)	6.36 (1.69, 23.89)
Birth weight	<4	106 (78.5)	252 (93.3)	358 (88.4)	1
	≥4	29 (21.5)	18 (6.7)	47 (11.6)	3.83 (2.04, 7.19)

Obstructed labor: attending physician diagnosed using American College of Obstetricians and Gynecologists (ACOG) obstructed labor as arrest of labor during the first and second stages of labor despite adequate uterine contraction because of mechanical obstruction manifested by signs of severe obstruction: caput and moulding formation, Bandl's ring, and edematous vulva.

**Table 4:** Bivariate and multivariable logistic regression analysis result of significant variables (*P* value < 0.05) at public hospitals in Tigrai, North Ethiopia.

Variables	Category	Cases	Controls	AOR (95% CI)
Age of mother	≤19	6 (4.4)	17 (6.3)	1
	20-34	95 (70.4)	211 (78.1)	1.37 (0.07, 44.8)
	≥35	34 (25.2)	42 (15.6)	3.09 (0.09, 104)
Ethnicity	Tigrai	130 (96.3)	267 (98.9)	1
	Afar	5 (3.7)	3 (1.1)	0.9 (0.61, 13.29)
Referral status	No	27 (20)	203 (70.4)	1
	Yes	108 (80)	67 (24.8)	7.29 (2.7, 19.68)^∗^
Antenatal follow-up	No	22 (16.3)	13 (4.8)	2.1 (1.35, 6.31)
	Yes	113 (83.7)	257 (95.2)	1
Number of antenatal visits	1	12 (10.7)	22 (8.3)	2.85 (1.02, 7.94)^∗^
	2-3	51 (45.5)	72 (27.3)	3.25 (0.63, 16.72)
	≥4	49 (43.8)	170 (64.4)	1
Parity	1-4	95 (70.4)	235 (87)	1
	≥5	40 (29.6)	35 (13)	1.84 (0.44, 7.69)
Gestational age in weeks	28-37	44 (32.6)	60 (22.2)	1
	37-42	82 (60.7)	198 (73.3)	1.08 (0.36, 3.25)
	≥42	9 (6.7)	12 (4.4)	0.71 (0.07, 6.82)
Previous history of C/S	No	109 (80.7)	263 (97.4)	1
	Yes	26 (19.3)	7 (2.6)	5.51 (0.85, 35.74)
Interpregnancy interval after C/S	Above 12 months	10 (7.4)	5 (1.9)	1
	Less than 12 months	16 (11.9)	2 (0.7)	3.42 (0.15, 79.46)
Pregnancy complicated by chorioamnionitis	No	131 (97)	269 (99.6)	1
	Yes	4 (3)	1 (0.4)	15 (0.16, 45)
Place of delivery	Health center	8 (5.9)	1 (0.4)	7.86 (0.57, 108.2)
	Hospital	127 (94.1)	269 (99.6)	1
Followed by partograph	No	118 (87.4)	61 (22.6)	10 (4.53, 19.24)
	Yes	17 (12.6)	209 (77.4)	1
Duration of labor	More than 18 hours	16 (11.9)	1 (0.4)	3.45 (2.12, 20.23)
	Less than 18 hours	6 (4.4)	197 (73)	1
Obstructed labor	No	55 (40.7)	242 (89.6)	1
	Yes	80 (59.3)	28 (10.4)	13.33 (4.23, 42.05)^∗^
Malpresentation	No	117 (86.7)	253 (93.7)	1
	Yes	18 (13.3)	17 (6.3)	1.59 (0.25, 10.23)
Labor started spontaneously	No	21 (15.6)	14 (5.2)	3.95 (0.93, 16.7)
	Yes	114 (84.4)	256 (94.8)	1
Trial of labor after caesarean delivery	No	122 (90.4)	267 (98.9)	1
	Yes	13 (9.6)	3 (1.1)	3.5 (0.1, 117.37)
Congenital anomalies of baby	No	126 (93.3)	267 (98.9)	1
	Yes	9 (6.7)	3 (1.1)	0.57 (0.057, 5.72)
Prehemoglobin level	≤7	15 (11.1)	19 (7)	2.03 (0.61, 6.82)
	7-11	54 (40)	27 (10)	1.72 (0.45, 6.55)
	≥12	66 (48.9)	224 (83)	1
Birth weight	<4	106 (78.5)	252 (93.3)	1
	≥4	29 (21.5)	18 (6.7)	5.68 (1.39, 23.2)^∗^

Abbreviations: C/S = caesarean section; ^∗^ = significant variables; AOR = adjusted odds ratio; COR = crude odds ratio.

**Table 5:** Maternal and fetal outcomes that develop uterine rupture among mothers who gave birth at public hospitals of Tigrai, North Ethiopia.

Variable	Category	Frequency (%)
What was done	Total abdominal hysterectomy	47 (34.8)
	Subtotal hysterectomy	28 (20.74)
	Uterine repair with bilateral tubal ligation	26 (19.25)
	Uterine repair without bilateral tubal ligation	34 (25.2)
Types of uterine rupture	Complete	75 (55.5)
	Incomplete	60 (45.5)
Location of uterine rupture	Anterior lower uterine segment	47 (34.8)
	Lateral uterine segment	35 (25.92)
	Posterior uterine segment	53 (39.25)
Excessive blood loss	Yes	78 (57.8)
Postoperative hemoglobin	<7 g/dl	48 (11.9)
	7-11 g/dl	34 (8.4)
	>11 g/dl	53 (13.1)
Blood transfusion	Yes	48 (11.9)
Registered maternal death	Yes	13 (9.6)
Immediate cause of maternal death	Hypovolemic shock	8 (2)
	Septic shock	3 (0.7)
	Others^∗^	2 (0.5)
Registered fetal asphyxia	Yes	34 (25.2)
Registered fetal death	Yes	101 (74.8)

^∗^Others = pulmonary edema and acute renal failure.

## Data Availability

The datasets used and/or analyzed during this study are available from the corresponding author on reasonable request.
